# Anteromedial knee osteoarthritis (AMOA) evaluated with magnetic resonance imaging (MRI): a cohort study of 100 patients

**DOI:** 10.1007/s00402-024-05511-2

**Published:** 2024-08-29

**Authors:** Kristine Ifigenia Bunyoz, Joseph Dixon, Jaison Patel, Anders Troelsen, Abtin Alvand, Will Jackson, Andrew Price, Nicholas Bottomley

**Affiliations:** 1 Department of Orthopaedic Surgery, Copenhagen University Hospital Hvidovre, Kettegård Alle 30, 2650 Hvidovre, Copenhagen Denmark; 2 Nuffield Orthopaedic Centre, Windmill Rd, Oxford, OX3 7LD UK; 3 Bart’s Bone and Joint Health, Royal London Hospital, Whitechapel Rd, London, E1 1FR UK

**Keywords:** Medial UKA, AMOA, Anteromedial knee osteoarthritis, Knee arthroplasty

## Abstract

**Introduction:**

Magnetic resonance imaging (MRI) scans are increasingly used for knee osteoarthritis evaluation and preoperative planning before unicompartmental knee arthroplasty (UKA), and often patients already have MRI scans before their initial surgeon consultation. This highlights the need for surgeons to understand anteromedial osteoarthritis (AMOA) patterns on MRI. Hence, we aim to describe MRI findings in patients with AMOA meeting current indications for medial UKA.

**Materials and methods:**

We analysed MRI scans from 100 knees evaluated for UKA between 2006 and 2013. Inclusion criteria comprised full-thickness medial compartment loss and intact lateral compartment joint space on preoperative radiographs. Assessment included cartilage lesions, osteophytes, meniscal damage, and anterior-cruciate ligament (ACL) status on tibial and femoral surfaces. Final decision to proceed with UKA relied on intraoperative findings, independent of MRI.

**Results:**

Complete anteromedial tibial and femoral cartilage loss preserved posterior cartilage rims was evident in all cases. Cartilage thinning occurred in the lateral compartment in 34% of cases. While 62% displayed lateral osteophytes, only 6 exhibited small areas of full-thickness cartilage loss. ACL abnormalities varied: 27% normal, 3% ruptured, and 70% had intrasubstance high signal. Larger osteophytes in the medial (p = 0.012) and lateral (p = 0.002) intercondylar notch correlated significantly with ACL damage. All underwent medial UKA, with no evidence of areas with full lateral compartment cartilage loss intraoperatively.

**Conclusions:**

The MRI findings confirmed the radiographic diagnosis of bone-on-bone medial disease but highlights a range of findings in the ACL, lateral compartment, and patellofemoral joint compartment for patients who met the current x-ray and intraoperative indication for UKA. Further research is required to understand if these MRI changes will affect long-term outcomes.

**Supplementary Information:**

The online version contains supplementary material available at 10.1007/s00402-024-05511-2.

## Introduction

The management of knee osteoarthritis (OA) continues to pose a substantial healthcare challenge, particularly given the lifetime susceptibility to developing symptomatic knee OA, which is estimated to exceed 40% in Western populations [1].

Within the domain of knee OA, distinct disease phenotypes emerge, characterized by varying patterns of articular deterioration within the knee joint. The comprehension, description, and recognition of these nuanced patterns of joint degeneration assume significance, as they have the potential to refine the precision of treatment strategies [2]. In orthopaedic surgery, the OA pattern often dictates the surgical treatment options available [3, 4]. Antero-medial osteoarthritis (AMOA) was initially described as a distinct OA phenotype by White and colleagues in 1991 [5]. AMOA involves medial compartment full-thickness cartilage loss with, preservation of the lateral compartment cartilage, and intact anterior cruciate ligament (ACL) [5]. Patients who present with this pattern of OA are candidates for unicompartmental knee arthroplasty (UKA) [6]. Today’s research shows that, in well-selected patients, UKA results in quicker recovery [7, 8], better kinematics, better range of movement, lower morbidity [9], lower mortality [10, 11], and better cost-effectiveness [12], compared to total knee arthroplasty (TKA). This has pushed research to extend the indications of medial UKA, to increase the number of patients benefitting from the procedure. Furthermore, it has been suggested that nearly half of patients considered for a knee replacement are suitable for a medial UKA [3].

The radiological features of AMOA have been well described using plain film radiographs [13, 14] and confirmed with histological evaluation [15]. However, since the introduction of magnetic resonance imaging (MRI), it has become a common method for researching and assessing knee OA radiologically. MRI is a non-invasive method that offers a comprehensive evaluation of the knee joint. It provides valuable benefits, such as the ability to assess soft tissues and offers cross-sectional imaging. So far, only one study has employed MRI to examine AMOA [16] and a detailed MRI description has not yet been reported. Furthermore, the use of MRI scans is on the rise in clinical practice and therefore a precise MRI-based depiction of AMOA holds significant value for both the research community and Orthopaedic surgeons, as a more comprehensive understanding of the disease pattern stands to enhance the ongoing refinement of patient selection, to help with preoperative planning and, consequently, lead to the improvement of outcomes following UKA.

Therefore, this paper aims to describe the MRI characteristics of end-stage AMOA in patients who meet current widely used indications for medial partial knee replacement. We will investigate the pattern of joint damage, including the state of the articular cartilage surfaces, and the anterior cruciate ligament.

## Patients and methods

### Design, setting, and participants

This study follows the strengthening the reporting of observational studies in epidemiology (STROBE) guideline. We retrospectively evaluated prospectively collected MRI data of 100 knees (90 subjects) who were assessed for possible UKA between 2006 and 2013 at the Nuffield Orthopaedic Center, United Kingdom. Patients were included if they met the preoperative radiographic criteria for partial knee replacement demonstrating full-thickness medial compartment loss and preservation of the lateral compartment on preoperative plain radiographs[5]. To understand the role of MRI in UKA, MRI was performed routinely on all subjects duringthis period to access the ACL, although this was not standard practice before UKA.. The decision to proceed with a UKA was based on intraoperative findings, not the MRI results. This decision was made to adhere to already established indications for UKAAll patients subsequently underwent partial knee replacement, with three having simultaneous ACL reconstruction.

### MRI evaluation and scoring

All MRI examinations were performed on a Phillips 3 Tesla imager. The image set included coronal, axial and sagittal fat-saturated proton density (FSPD) images (TR = 4000 m/s, TE = 40–50 ms, 256 × 256 matrix, FOV 14 cm, NEX 2) and Sagittal T1 weighted repolarised DRIVE sequence (TR = 630, TE = 20 ms, 256 × 256 matrix, FOV 14 cm, NEX 2). The MRI scans (89/100 scans) were scored independently by a Consultant Musculoskeletal Radiologist and a Clinical Research Fellow, using an agreed MRI atlas. Both readers scored all images independently. In case of any disagreement, images were reviewed, and consensus opinions were reached. The tibial and femoral joint surfaces of the medial, lateral, and patellofemoral compartments of the knee were assessed for the presence of full-thickness cartilage lesions, osteophytes, and subchondral bone signal changes. Meniscal damage and ACL status were also assessed.

### Anterior collateral ligament

The ACL was scored from 0 to 3, with 0 being normal, 1 when minor signal changes were present in an intact ligament, 2 with more significant splaying, kinking or ganglion formation but with preserved orientation of fibres and 3 indicating rupture (Appendix 1e in ESM). MRI scans with scores of 2 and 3 were compared to preoperative lateral X-rays for signs of ACL damage. This comparison followed the method described by Hamilton et al. [17], where an abnormal or absent ACL is indicated by tibial erosion extending to the back of the tibial plateau, sometimes accompanied by posterior femoral subluxation.

### Cartilage and bone marrow lesions

When assessing cartilage lesions and bone marrow lesions, the tibial and femoral sides of each compartment were scored separately but were not further sub-divided. The trochlea (femoral side of patellofemoral joint) was defined as the area of femoral articular surface anterior to a line passing from the femoral mid-condylar point to the most anterior aspect of the meniscus in the sagittal plane (Appendix 1a in ESM). The articular surface posterior to this line was considered part of the tibiofemoral joint. A subchondral bone marrow lesion was defined as an area of high signal abutting the subchondral plate on any one of the FSPD images.

### Osteophytes

Osteophytes were assessed in eight locations: the joint line in each of the four tibiofemoral compartment margins, on each side of the intercondylar notch and the posterior margin of each femoral condyle. The posterior femoral osteophytes are found at the medial and lateral margins of each posterior femoral condyle, and we call these the “mouse ear” osteophyte (Appendix 1b in ESM). They are most visible on the axial view and are considered important clinically because inadequate clearance may lead to technical difficulties with prosthetic joint placement. Notch osteophytes were assessed on the coronal view. The osteophytes were scored on a scale of 0–3 with 0 being a normal smoothly curved margin from the subchondral plate to the tibial or femoral rim with only one change in angle and with no overlap over a line drawn vertically from the tibial or femoral rim (Appendix 1c in ESM). A score of 1 was a minor deviation from the above, 2 an obvious and 3 a large irregularity.

### Meniscus extrusion and tear

The meniscus was assessed for extrusion and tears. A tear was defined as signal change within the meniscus that extended to either the femoral or tibial joint surface. This definition has been used in previous epidemiological assessments of the meniscus [18]. Extrusion was defined as more than 30% of the meniscal width outside a vertical line up from the tibial rim (Appendix 1d in ESM) [19]. When the meniscus was seen to almost be grossly abnormal and almost absent, with no normal anatomy to define tears, it was classified as macerated.

### Intraoperative assessment

All surgeries were performed by a lead surgeon experienced in performing UKA. Following a long-established protocol in the department, all knees were assessed intraoperatively for ACL function, bone on bone in the medial compartment, the lateral facet of the patellofemoral joint, and the state of the cartilage in the distal weight-bearing surface of the lateral compartment. Following the standard Oxford indications non-weight bearing lateral compartment changes and focal minor macroscopic damage on the weight-bearing surface were not a contraindication to UKA. In cases where larger areas of full-thickness cartilage loss were found in the weight-bearing part, a conversion to TKA was standard practice. However, as not all parts of the lateral compartment can be inspected intraoperatively areas of full-thickness loss may go undetected during surgery. The final decision to perform a medial UKA did not depend on the MRI scan but solely on the intraoperative findings.

### Statistics

Frequencies were reported as percentages. Descriptive results were described as means with standard deviations (sd) or medians with interquartile ranges (IQR) depending on data distribution. Cohen’s kappa value was calculated to assess intra- and interobserver agreement in 89/100 MRI scans. Fishers exact test and chi squared test were used in comparison of categorical data. Significance was set as p < 0.05. All analyses were performed in R (R version 4.2.2 (2022-10-31)).

### Ethics

This study was carried out in accordance with the world medical association of Helsinki. The study was registered as a service evaluation and quality improvement.

## Results

### Patient demographics

52% of patients were females and 49% of the cases were performed on the right side. Mean age at the time of the MRI scan was 60.6 years (sd 9.03).

### Medial compartment changes on MRI

All medial compartments showed full thickness cartilage loss both in the anteromedial tibial cartilage and the femoral cartilage. In all cases there was an obvious rim of retained cartilage on the posterior rim of the medial compartment. The medial meniscus was extruded or teared in 98% of cases with 41% being macerated. On the medial side there was a common pattern of osteophyte formation; 93% presented with an osteophyte on the posteromedial and posterolateral aspect of medial femoral condyle (mouse ear osteophyte); 92% presented with a medial tibia osteophyte; 91% with a medial femoral osteophyte, and 85% with an osteophyte in the intercondylar notch.

### Anterior cruciate ligament changes on MRI

**Fig. 1:**
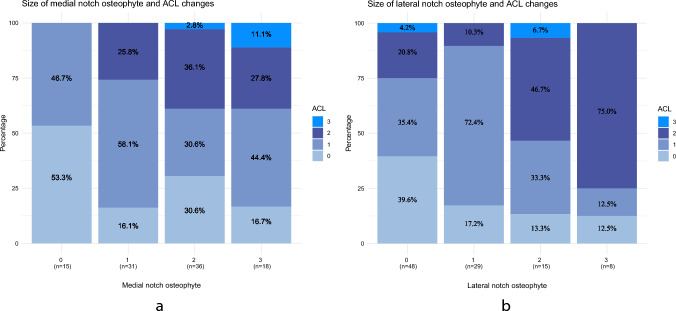
**a/b** Stacked bar plot of medial (**a**) and lateral (**b**) notch osteophytes and ACL changes on MRI. Illustrates the association between the severity of medial (**a**)/lateral (**b**) notch osteophyte and the presence of ACL changes on MRI. The x-axis displays categories representing the severity of the medial (**a**)/lateral (**b**) notch osteophytes, while the y-axis quantifies the percentage of occurrences. Each bar is divided into segments of different colors, each corresponding to a level of ACL changes. Osteophyte severity scoring; 0 = no osteophyte, 1 = a minor deviation, 2 = moderate deviation, 3 = large irregularity. ACL scoring system; 0 = normal, 1 = minor signal changes present in an intact ligament, 2 = more significant splaying, kinking or ganglion formation but with preserved orientation of fibres, 3 = rupture

On assessment of the ACL 3% showed rupture and 70% showed intrasubstance high signal (44% grade 1 and 26% grade 2) and 27% were normal. There was a significant relationship between increasing size of osteophyte in both the medial (p = 0.0124) and lateral (p = 0.0019) intercondylar notch and increasing damage to the ACL (Fig. 1a, b). The MRI findings for patients with either an ACL rupture or a grade 2 intrasubstance high signal were directly compared to preoperative lateral view X-rays. The goal was to determine if any signs of ACL damage could have been detected on the X-rays (Table 1).

**Table 1:** Anterior-cruciate ligament changes on MRI scan and corresponding x-ray findings

ID	Age at surgery	Gender (remove)	ACL rupture (3) or grade 2 intrasubstance high signal (2) on MRI	Lateral view X-ray changes indicating ACL damage
1	50	M	3	Yes
2	46	M	3	Yes
3	53	F	3	Yes
4	65	M	2	No
5	67	F	2	No
6	67	M	2	No
7	67	M	2	No
8	59	M	2	No
9	73	M	2	No
10	67	M	2	No
11	61	M	2	No
12	61	F	2	No
13	69	F	2	No
14	75	M	2	No
15	61	F	2	No
16	63	M	2	No
17	62	M	2	No
18	59	M	2	No
19	56	M	2	No
20	54	M	2	No
21	68	M	2	No
22	48	M	2	No
23	61	F	2	No
24	66	F	2	No
25	49	M	2	No
26	53	M	2	No
27	65	M	2	No
28	64	M	2	No
29	62	F	2	No

### Lateral compartment changes on MRI

34% of the cases showed either areas of partial or full-thickness cartilage thinning on the MRI scans. 25% of the lateral femoral and 13% of the lateral tibial surfaces had some partial cartilage thinning in the central weight-bearing area. Small areas with full-thickness cartilage loss in the central area of the lateral compartment was seen in 3 cases on the femoral side and 5 cases on the tibial side. These small areas of full-thickness loss were not considered a contraindication to UKA intraoperatively and therefore all the patients received a medial UKA at the time of surgery. The MRI findings for 34 patients showing lateral compartment changes were directly compared to preoperative lateral view X-rays. The goal was to determine if any signs of these cartilage changes could have been detected on the X-rays (Table 2).

**Table 2:** Lateral compartment cartilage changes on MRI and corresponding x-rays

ID	Age at surgery	Gender	Small area with partial (1) or full-thickness (2) cartilage loss on MRI	X-ray changes in the lateral compartment (KL-grade)
1	56	M	2	0
2	47	F	2	0
3	67	F	2	0
4	61	F	2	0
5	74	M	2	0
6	49	M	2	0
7	53	M	2	0
8	75	M	1	0
9	58	M	1	0
10	76	M	1	0
11	67	M	1	0
12	46	M	1	0
13	51	F	1	0
14	69	F	1	0
15	72	F	1	0
16	61	F	1	0
17	56	M	1	0
18	59	M	1	0
19	56	M	1	0
20	79	F	1	0
21	54	M	1	0
22	57	F	1	0
23	53	F	1	0
24	61	F	1	0
25	66	F	1	0
26	61	F	1	0
27	73	M	1	0
28	55	M	1	0
29	73	M	1	0
30	79	F	1	0
31	65	M	1	0
32	64	M	1	0
33	44	F	1	0
34	62	F	1	0

**Fig. 2:**
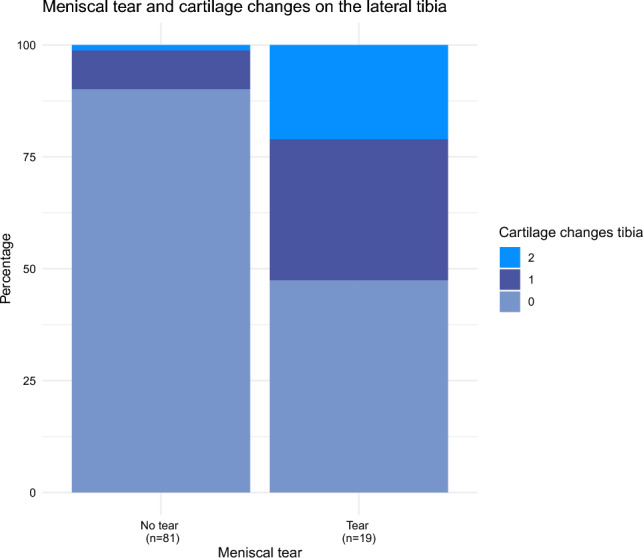
Stacked bar plot of lateral meniscal tear and cartilage changes on the lateral tibia. Illustrates the association between lateral meniscal tear and the presence of overlying cartilage changes on the tibia. The x-axis displays the presence of meniscal tear, while the y-axis quantifies the percentage of occurrences. Each bar is divided into segments of different colors, each corresponding to a level of cartilage changes. Cartilage change scoring; 0 = no change, 1 = area with partial thickness loss, 2 = area with full thickness loss

Seven percent had bone marrow lesions (BML) in the lateral distal femur, 12% in the proximal tibia, however, none of these cases showed any areas of full-thickness cartilage loss in either the femur or the tibia. There was however a significant relationship between the presence of BML and overlying cartilage thinning in the lateral compartment on the femur (p = 0.028). Assessment of the lateral meniscus showed that 19% were torn and 2% were extruded. There was a significant relationship between lateral meniscal tear and lateral cartilage thinning (p = 0.009), with the association stronger still when assessing the lateral tibial cartilage in isolation (p =  < 0.0001) (Fig. 2). Whilst 62% of subjects were seen to have a lateral tibiofemoral osteophyte, only 7 of these had a small area on the MRI scan with full thickness cartilage loss on either the femur or the tibia.

### Patellofemoral joint changes on MRI

At least a small area of full-thickness cartilage loss in either the patella or trochlea surface was seen on the lateral side in 35% of cases and on the medial side in 29% of cases. 8% of all knees had some full thickness loss in both the medial and lateral aspects of the PFJ. The PFJ cartilage was normal in 18% of cases.

### Intraoperative findings

Intra-operative findings revealed anteromedial OA with bone-on-bone in the medial compartment and an intact ACL in 97 cases (97%). 3 patients had a ruptured ACL (already identified on the MRI scan) which was re-established intraoperatively. None of the patients presented with macroscopically visibly full cartilage loss on the weight bearing part of the lateral compartment. All the patients with an intact anterior cruciate were deemed to meet the accepted indications for medial UKA and went on to receive this treatment. The three patients who did not meet the accepted indications due to an absent ACL all underwent medial UKA with simultaneously ACL reconstruction.

## Discussion

In summary, the MRI scans revealed full-thickness cartilage loss in the medial compartment, mostly with retained posterior cartilage. ACL injuries were present in 3%, and the medial meniscus was frequently extruded or torn (98%). Osteophytes were common, with significant associations between the size of notch osteophytes and ACL damage. We identified common patterns of osteophytes with most patients having significant posteromedial and posterolateral osteophytes. In the lateral compartment, 34% displayed cartilage thinning, with some full-thickness losses. Bone marrow lesions correlated with overlying lateral cartilage thinning on the femur, but not on the tibia. Also, lateral meniscal tears were associated with lateral cartilage thinning. Only 18 of the cases showed completely normal patellofemoral joint cartilage.

The increasing use of MRI scans in daily clinical practice, to improve preoperative assessment, patient selection, surgical planning, and outcome prediction, is forcing orthopaedic surgeons to evaluate the findings of MRI scans to be able to recognize different patterns of knee OA. Understanding the AMOA pattern on MRI scans is therefore important for making informed decisions about offering UKA to suitable patients. However, the literature describing AMOA on MRI is sparse and it is not clear how the extra knowledge gained from MRI scans should be interpreted in a clinical setting. In our study, the ACL was rarely normal at MRI and 3 cases showed a ruptured ACL. The 3 cases of ACL rupture were confirmed intraoperatively, and an ACL reconstruction was performed simultaneously with the medial UKA. The rest of the cases were found with functionally intact ACL intraoperative despite MRI scans demonstrating intrasubstance high signal (44% grade 1 and 26% grade 2). The mismatch between the intrasubstance high signal and a functionally intact ACL found intraoperatively was also found by Sharpe et al.[16]. However, currently, the clinical significance of high signal within an otherwise functionally intact ACL remains unknown. We do not know how these findings may affect the long-term outcome for this patient group, and this should be investigated in long-term follow-up studies comparing outcomes between UKA patients with normal ACL signals and those with intrasubstance high signals on preoperative MRI scans. Looking back on the preoperative X-rays, we could not refind any signs indicating the altered signal in the ACL on the preoperative lateral view X-rays, except for one of the cases with a ruptured ACL. So adhering to the current indications for a UKA, we suggest that the the best radiological method of determining the clinical ACL function on MRI might be to look for a clear continuity of fibres between origin and insertionin association with intact posterior tibial cartilage on the medial side, even if the fibers are disrupted by ganglionic material-. The MRI scan could therefore potentially be useful preoperatively in determining the state of the ACL and to prepare the surgeon for a possible conversion to a TKA or a co-performance of an ACL reconstruction.

In the medial compartment the meniscus was always extruded or macerated. It is known that both meniscal tears and maceration are associated with increased cartilage loss in the same compartment [18] and our findings in the medial compartment reinforce this. There continues to be debate as to the timing of the meniscal extrusion in the development of AMOA. However, it has been suggested that meniscal extrusion likely precedes cartilage loss in the corresponding compartment [20].

19% of lateral menisci were torn and there was a significant relationship between this meniscal damage and lateral compartment cartilage thinning. As in the medial joint, the temporal relationship between meniscal tear and cartilage volume loss is unknown. Bone marrow lesions in the lateral compartment should not be taken as a surrogate marker of full-thickness cartilage loss since 7% of lateral femora and 12% of lateral tibiae had a BML, none of which were associated with full-thickness cartilage loss.

Tibiofemoral osteophytes in the lateral compartment were common (62% of all subjects) however only 7 cases had co-existing small areas with full thickness lateral compartment cartilage loss on the MRI scan. Lateral osteophytes can therefore not be taken as a surrogate marker for full-thickness cartilage loss and care should be taken when interpreting plain radiographic images of the knee joint.

It is stated that up to 50% of the patients presenting with osteoarthritis, are candidates for a medial UKA [3]. However, the current use of UKA in the UK (2022) is only 13% [21]. One of the commonest modes of failure is lateral progression [4, 22] and if UKA is to become more common, an improved understanding of the risk factors for lateral progression is important. Whilst there were no cases with large areas of macroscopically full-thickness cartilage loss in weight-bearing areas intraoperatively in the lateral compartment, this study identifies a sub-group of patients with abnormal MRI cartilage findings in the lateral compartment (34%). This finding is supported by Disler et al. [23] who described lateral compartment changes on MRI to be 2–3 times higher than those detected byarthroscopy. However, similar to the ACL changes, the clinical significance of these lateral MRI findings regarding long-term surgical outcomes and progression of lateral OA is not well understood. One study has looked at the correlation between abnormal preoperative MRI scans and failure of UKA, but it had a limited sample size of 44 MRI scans and did not differentiate the location of the abnormalities [24]. Another study has compared the functional outcomes after medial UKA in patients with and without lateral meniscal abnormalities on MRI and found no difference between group [25]. Standard assessment of lateral compartment changes before medial UKA is usually performed with valgus stress radiographs. However, a recent study found MRI to be excellent in evaluating lateral weight-bearing cartilage injuries before medial UKA compared to stress radiographs [26].

Our study confirms that varying amounts of patellofemoral cartilage damage, from normal to full thickness loss in both facets, are seen in patients with antero-medial OA. Whilst there is evidence that moderate cartilage damage in the PFJ does not affect outcome of UKA [27], it remains an important factor for many surgeons in assessing the joint prior to arthroplasty as it is often used as a contraindication to UKA [28].

Despite the additional knowledge gained from MRI, its clinical significance remains unclear without long-term follow-up data on patients with these MRI changes. To truly understand how MRI findings impact patient outcomes after UKA, further long-term studies are necessary. These studies should focus on comparing outcomes between UKA patients with normal MRI findings and those with high intrasubstance signals, lateral cartilage changes, or other abnormalities.

This study is not without limitations. We have developed a series of acquisition sequences that will identify most OA changes, but some detail of knee pathology may be lost due to our chosen slice thickness. Decreasing slice size will increase the accuracy of finding changes but may decrease the cost-effectiveness of the investigation. We believe our protocol is a satisfactory compromise. The integrity of the most peripheral cartilage in the region around the tibial spine and the very periphery of the femoral condyles was not specifically assessed. Whilst the kappa assessment is valuable for assessing reproducibility of categorical data, when used to assess agreement over so many variables, as is the case in this paper, the value is often low. However, we believe our reported value of 0.63 was satisfactory. As a comparison to the MRI findings intraoperative assessment of the ACL and the cartilage in the lateral compartment were performed, as these structures are both important factors for outcome after medial UKA. However, this study was not designed to enable a detailed correlation of the MRI grades of ACL damage and the histopathological properties of the ACL. The significance of an increased MRI signal in the ACL on the specific structural properties of the ACL is therefore not answered. However, despite these limitations this study provides an important description of the pattern of AMOA on MRI scans, which can be useful to the orthopaedic surgeons in their process of selecting patients for medial UKA.

In conclusion, this study has identified the MRI scan findings associated with anteromedial OA. The findings confirmed the radiographic diagnosis of bone-on-bone medial disease but highlights a range of findings in the ACL, lateral compartment, and patellofemoral joint compartment for patients who met the intraoperative indication for partial knee replacement. In particular, (1) we identified that isolated high signal in the intact and functioning ACL is common, (2) that patients on macroscopic assessment of the lateral compartment who meet the current criteria for partial knee replacement may have cartilage change on the MRI scan, (3) in one third of patients articular cartilage damage was identified on the lateral facet of the patellofemoral joint. At present these MRI characteristics are not considered contraindications. MRI scans can provide valuable information on the assessment of anteromedial OA. Further research is required to understand if these changes will affect long-term outcome.

## Supplementary Information

Below is the link to the electronic supplementary material.

Supplementary file1 (DOCX 829 KB)

## Data Availability

The data that support the findings of this study are available from Nuffield Orthopaedic Center, but restrictions apply to the availability of these data, which were used under license for the current study, and so are not publicly available. Data are however available from the authors upon reasonable request and with permission of Nuffield Orthopaedic Center.
