# Supplementary material for: Anteromedial knee osteoarthritis (AMOA) evaluated with magnetic resonance imaging (MRI): a cohort study of 100 patients
Source: Arch Orthop Trauma Surg. 2024 Aug 29;144(8):3439–47. doi: 10.1007/s00402-024-05511-2 (PMC11417064; doi:10.1007/s00402-024-05511-2)
Supplement: Supplementary file 1 — Supplementary file1 (DOCX 829 KB) [file 402_2024_5511_MOESM1_ESM.docx]

**Appendix 1**

1a: Patellofemoral and tibiofemoral joint regions


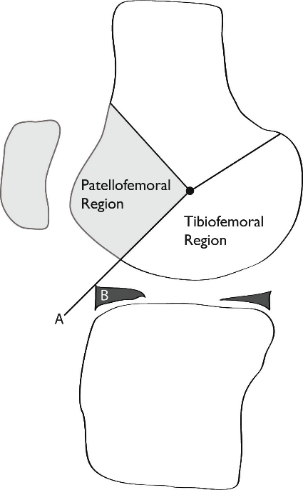


Diagram illustrating the division between the patella-femoral and tibiofemoral joint regions (line A) using the anterior margin of the meniscus in the sagittal plane as a landmark (B).

1b: The “mouse ear” osteophyte


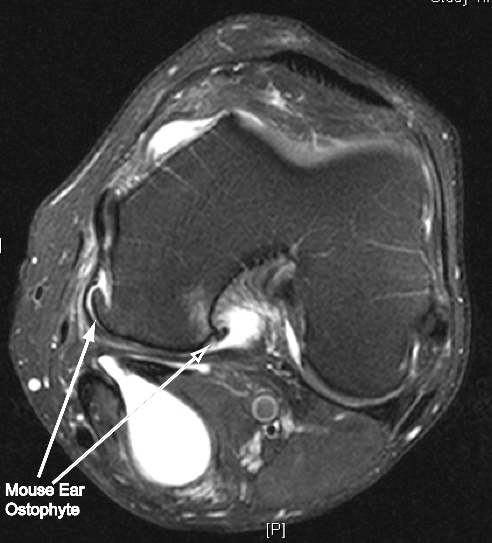


The ‘”mouse ear”osteophyte is found at the medial and lateral margins of each posterior femoral condyle

1c: Osteophyte severity


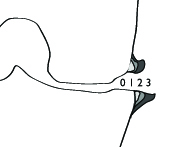


Illustration of osteophyte severity scoring; 1 = a minor deviation, 2 = moderate deviation, 3= large irregularity

1d: Meniscal extrusion


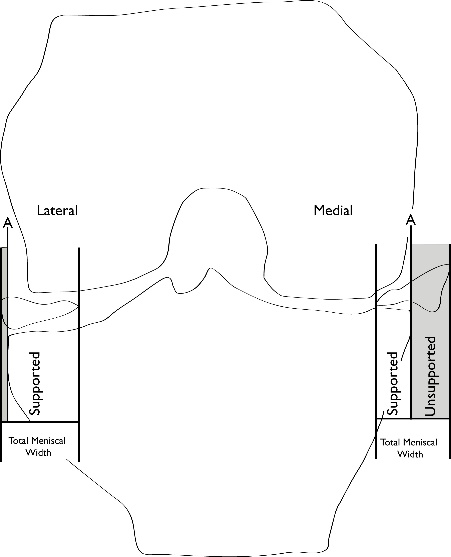


Illustration of meniscal extrusion; extrusion was defined as more than 30% of the meniscal width lying outside a vertical line up from the tibial rim

1e: ACL scoring system


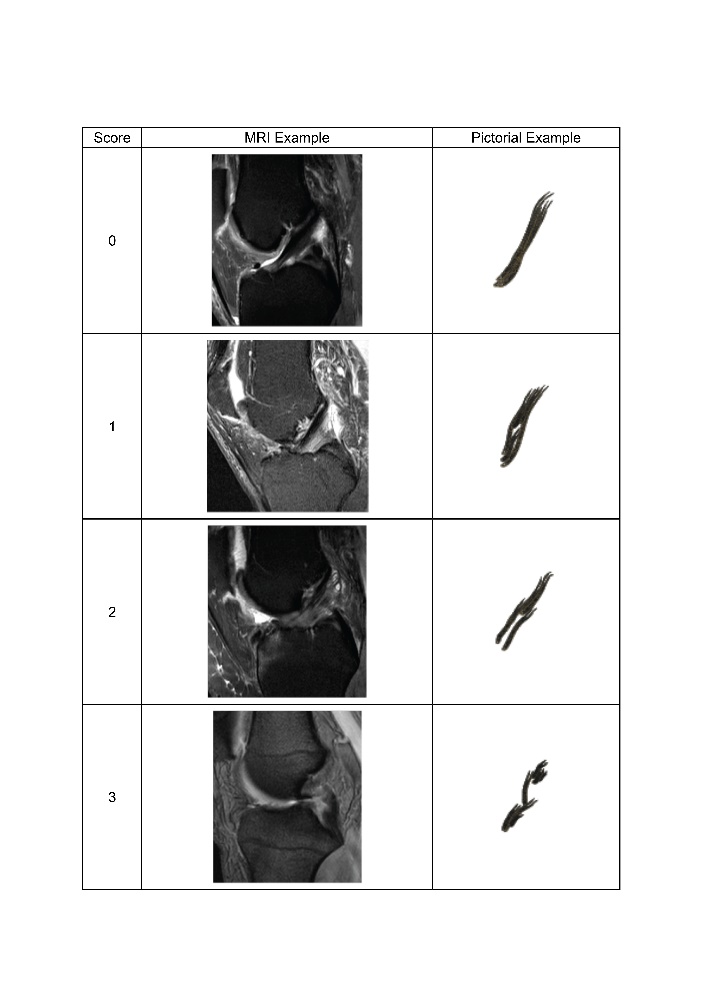


Illustration of ACL scoring system; 0= normal, 1= minor signal changes present in an intact ligament, 2 = more significant splaying, kinking or ganglion formation but with preserved orientation of fibres, 3= rupture
